# Fibrinolytic System and Cancer: Diagnostic and Therapeutic Applications

**DOI:** 10.3390/ijms22094358

**Published:** 2021-04-22

**Authors:** Niaz Mahmood, Shafaat A. Rabbani

**Affiliations:** 1 Department of Medicine, McGill University, Montréal, QC H4A3J1, Canada; niaz.mahmood@mail.mcgill.ca; 2 Department of Medicine, McGill University Health Centre, Montréal, QC H4A3J1, Canada

**Keywords:** uPA, uPAR, PAI-1, PA system, cancer

## Abstract

Fibrinolysis is a crucial physiological process that helps to maintain a hemostatic balance by counteracting excessive thrombosis. The components of the fibrinolytic system are well established and are associated with a wide array of physiological and pathophysiological processes. The aberrant expression of several components, especially urokinase-type plasminogen activator (uPA), its cognate receptor uPAR, and plasminogen activator inhibitor-1 (PAI-1), has shown a direct correlation with increased tumor growth, invasiveness, and metastasis. As a result, targeting the fibrinolytic system has been of great interest in the field of cancer biology. Even though there is a plethora of encouraging preclinical evidence on the potential therapeutic benefits of targeting the key oncogenic components of the fibrinolytic system, none of them made it from “bench to bedside” due to a limited number of clinical trials on them. This review summarizes our existing understanding of the various diagnostic and therapeutic strategies targeting the fibrinolytic system during cancer.

## 1. Fibrinolysis and Fibrinolytic System: An Introduction

The association between fibrinolysis and cancer has been known for more than a hundred years. In the early part of the 20th century, it was reported that tumor tissues possess fibrinolytic properties [1,2]. Fischer observed that explants of tumors obtained from viral sarcomas in chickens could cause the breakdown of blood clots, whereas the explants obtained from normal connective tissue lacked such activity [3]. However, the importance of fibrinolysis during cancer progression remained underappreciated until the early 1970s, when the involvement of a proteolytic enzyme that increases fibrinolysis during oncogenic transformation was described [4,5]. Since then, understanding the biology of fibrinolysis during cancer progression as well as its therapeutic targeting has gained much attention. The increase in fibrinolytic activity in the tumor is primarily attributed to the plasminogen activators (PA) secreted by the tumors [6], and therefore the fibrinolytic system is interchangeably designated as the PA system in cancer. It is now well established that several components belonging to the fibrinolytic system are deregulated in cancer [7]. 

In normal physiological conditions, fibrinolysis refers to the enzymatic degradation process of the fibrin mesh of blood clots and is facilitated by the components of the PA system including the key protease plasmin, its precursor inactive plasminogen, and activators tissue- and urokinase-type plasminogen activators (tPA and uPA) [7]. Plasmin is primarily derived from inactive plasminogen by the action of either tPA or uPA. Once activated, plasmin degrades the accumulated fibrin into soluble fibrin degradation products (FDP). Plasmin activities are balanced by plasmin inhibitors α2-antiplasmin (α2-AP) and α2-macroglobulin to counteract the free plasmin concentration [8,9]. On the other hand, plasminogen activator inhibitor-1 (PAI-1) and -2 (PAI-2) and activated protein C inhibitor (PAI-3) modulate the function of plasminogen activators (tPA or uPA) [7,8,10,11]. The components of the fibrinolytic system play crucial roles in various other biological processes that include cell migration, tissue remodeling, modulation of various growth factors and cytokines, regulation of immune response, and chemotaxis [12,13,14,15,16].

Plasmin can directly deregulate the fibrinolytic system in various pathological conditions including cancer; however, therapeutic targeting of plasmin is not always a feasible option in clinical settings. This is due to the role of plasmin in crucial physiological processes such as tissue remodeling and thrombolysis [10], and blocking plasmin activity may lead to systemic disorders. Ploplis et al. demonstrated that animals with homozygous deletion of the plasminogen (*Plg^−/−^*) gene showed growth retardation and reduced fertility and survival compared to the wildtype (*Plg^+/+^*) group [17]. However, targeting the molecular factors (tPA, uPA, uPAR, and PAI-1) that are upstream of plasminogen did not impair normal growth characteristics of the animals. While there is strong evidence suggesting the involvement of uPA, uPA receptor (uPAR), and PAI-1 in various stages of cancer growth and progression, tPA is less commonly associated with cancer [7]. For example, Shapiro et al. showed that depletion of uPA inhibits tumorigenesis in a rodent model of melanoma by limiting the availability of critical growth-promoting factors such as the basic fibroblast growth factor (bFGF) in the tumor microenvironment [18]. Loss-of-function assays against uPA and uPAR genes increased apoptosis of cancer cells [19,20]. Furthermore, the uPA/uPAR/PAI-1 axis has been shown to play a critical role in mediating angiogenesis and metastasis in different types of cancer via the modulation of several well-known cancer-related signaling pathways such as the Ras/Raf/MEK/ERK and PI3K/AKT pathways [20,21,22,23]. Therefore, attempts to pharmacologically target uPA, uPAR, and PAI-1 have been made in the case of almost all types of cancer. For the rest of the review, we will therefore focus on these three components (uPA, uPAR, and PAI-1) of the fibrinolytic system and describe the advances made to target them in cancer therapeutics and diagnostics.

## 2. Components of the Fibrinolytic System as Diagnostic Biomarkers

Due to the extensively described multifaceted role of the components of the fibrinolytic system during different stages of cancer progression, various efforts have been conducted for their use in diagnostic approaches [9]. Some key examples of the potential use of the fibrinolytic components in cancer diagnosis are described below.

### 2.1. uPA and PAI-1 as Cancer Biomarkers

Around three decades ago, Duffy et al. first described that breast cancer patients showing a higher activity of uPA had a significantly shorter disease-free interval compared to those with lower uPA activity [24]. Later on, Jänicke et al., in two separate studies, reported on the elevated levels of uPA and PAI-1 proteins in primary breast tumors, which correlated with the poor prognosis of the patients [25,26]. Using data from 8377 breast cancer patients, it was further confirmed that the higher levels of uPA and/or PAI-1 in breast tumors correlate with the aggressiveness of cancer and poor relapse-free and overall survival of the cancer patients [27]. The clinical utility of uPA and PAI-1 as biomarkers has been demonstrated by two separate level-of-evidence-1 studies [28,29]. Several commercially available enzyme-linked immunosorbent assay (ELISA) kits have been developed for detecting the levels of uPA and PAI-1 proteins [30]. The clinical utility of one of the commercially available ELISA kits, FEMTELLE^®^, was further validated by a multicenter external quality assessment (EQA) program [31]. The results from six independent laboratories showed that the coefficient of variation (CV) to detect uPA and PAI-1 by using the FEMTELLE^®^ kit ranged between 6.2–8.2% and 13.2–16.6%, respectively [31,32]. The American Society of Clinical Oncology (ASCO) recommended using an ELISA test to measure uPA and PAI-1 levels as prognostic markers for assessing the risk of breast cancer and a predictive marker to determine the suitable adjuvant therapies for the patients [33]. One of the major caveats of ELISA-based assays is the requirement of either fresh or fresh-frozen tissue samples, which is logistically challenging [34]. Therefore, the use of alternative methods without the need for freshly processed samples to determine the uPA and PAI-1 levels has been explored. The utilization of formalin-fixed paraffin-embedded tissues to assess uPA and PAI-1 seems to be the most straightforward solution; however, the presence of uPA and PAI-1 antigens in both the tumor and stroma cells makes consistent immunohistochemical scoring very challenging [34]. With the emerging use of machine learning algorithms in various aspects of biology, it would be interesting if artificial intelligence (AI) technology can be used in this regard to automatically distinguish the tumor and surrounding stromal tissue and thereby solve a long-standing biological problem.

Several groups have attempted to measure uPA (also known as the *PLAU* gene) and PAI-1 (also known as *SERPINE1*) mRNAs in cancer through quantitative reverse transcription-polymerase chain reaction (qRT-PCR) [35,36] and nucleic acid sequence-based amplification (NASBA) assays [37]. Even though the use of the mRNA-based approach does not require fresh/fresh-frozen tissues, the incongruities between the studies to quantify and use the uPA and PAI-1 mRNAs as biomarkers for cancer patients prevented their use in clinical settings [34]. The recent advancements in sequencing technologies may overcome the cross-laboratory discrepancies in mRNA measurement, and targeted sequencing of uPA and PAI-1 mRNA may provide concrete evidence as to whether they can be used as biomarkers for cancer patients.

Epigenetic modification through DNA methylation provides an additional layer of transcriptional regulation of gene expression [38]. Anomalous DNA methylation is a hallmark of cancer [38], and DNA methylation-based biomarkers are emerging as promising frontiers for cancer diagnosis [39]. The higher stability of DNA, as well as its methylation marks and the fact that it can be efficiently isolated from formalin-fixed paraffin-embedded tissue samples, makes it well suited for use as diagnostic biomarkers [34]. Our group was the first to test and report the alteration in the status of uPA promoter methylation in cancer cells [40]. We further demonstrated an inverse association between uPA promoter DNA methylation and its mRNA expression as the tumor progresses to a more clinically aggressive grade [41]. uPA promoter methylation is decreased as the cancer becomes more aggressive, which indicates that the assessment of uPA promoter methylation may serve as an early diagnostic marker. A similar inverse relationship between mRNA expression and promoter DNA methylation has been demonstrated in the case of the PAI-1 gene [42]. DNA methylation assays are relatively simple to develop [39], and the advent of targeted sequencing technologies made it easier to assess the methylation sites on a specific location of the genome. Further studies on uPA and PAI-1 promoter methylation using larger cohorts of cancer patients belonging to different demographics are warranted to confirm their prognostic significance in cancer diagnosis. 

### 2.2. uPAR as a Diagnostic Biomarker

Among the various members of the fibrinolytic system, uPAR holds a dominant position in terms of its applicability in cancer diagnosis and therapeutics. This is because of the fact that uPAR is scarcely present in healthy tissues, but its levels are elevated in malignancies where it is often associated with the aggressiveness of the cancer [9,43]. These characteristics of uPAR make it an ideal candidate for non-invasive imaging for cancer diagnosis and response to therapy. Almost two decades ago, we had shown that when an anti-rat uPAR antibody radiolabeled with ^125^I was inoculated into animals with pre-established prostate and mammary tumors, an increase in radioactivity was determined in the primary tumors as well as various metastatic sites [44]. However, the rats receiving control IgG antibody radiolabeled with ^125^I showed minimum radioactivity. This study suggested that uPAR imaging can be used for cancer diagnosis. 

Various attempts have been made by different groups to utilize uPAR for cancer diagnosis. A small molecule uPAR binding peptide called AE105 in conjugation with ^64^Cu-labeled DOTA was evaluated for positron-emission tomography (PET)-based molecular imaging [45,46]. In 2015, Persson et al. reported on the utilization of AE105 for the first ever uPAR PET scanning in humans [47]. Several other groups are using a uPAR-based imaging strategy to determine the aggressiveness of cancer in humans, and there are more than ten clinical trials that are either ongoing or have recently been completed. Some of the ongoing clinical trials include NCT02965001 (for head and neck cancer), NCT03307460 (for prostate cancer), NCT03278275 (neuroendocrine tumors), and NCT02755675 (malignant pleural mesothelioma, non-small cell lung cancer, and large cell neuroendocrine carcinoma of the lung). 

Antibodies targeting uPAR (such as ATN-658, 2G10) and uPA (ATN-291) were also utilized for cancer imaging [48,49,50]. One of the major advantages of using antibodies for oncological imaging is that they possess a relatively longer half-life in the serum compared to peptide-based agents, thereby prolonging the timeframes for cancer imaging up to days [47,51]. On the other hand, the half-life of peptide-based agents such as AE105 is shorter, and the imaging timeframes may last several hours only [47]. Yang et al. targeted uPAR imaging through conjugation of the amino-terminal fragment (ATF) of uPA with magnetic iron oxide nanoparticles (ATF-IO) for imaging mammary tumors in vivo [52]. In summary, regardless of the strategies used, uPAR-based oncological imaging holds great promise for cancer diagnosis. 

### 2.3. suPAR as a Cancer Biomarker

Another important avenue that holds great promise but needs more exploration is using soluble urokinase plasminogen activator receptor (suPAR) from body fluids as a biomarker of cancer. The plasma levels of suPAR are elevated in different types of pathological conditions such as inflammation, autoimmune diseases, virus infection, and chronic kidney diseases, where they serve as plasma biomarkers [53,54,55,56]. Rovina et al. demonstrated that suPAR can be potentially used as an early marker of respiratory failure in patients suffering from COVID-19-related pneumonia [57]. Elevated levels of suPAR were found in different types of cancer including colon, lung, prostate, breast, and ovarian cancers [53,58,59,60,61].

There two main forms of suPAR in the circulation: (i) the full-length suPAR, and (ii) the cleaved soluble uPAR (containing the D2 and D3 domains of uPAR) [62]. Due to the lack of the N-terminal domain, the cleaved form of suPAR cannot bind to most of the uPAR ligands, with the exception of formyl peptide receptor (FPR)-like 1 and 2 that do not require the presence of the D1 domain [62,63]. Therefore, the full-length suPAR is also known as the active form of suPAR and is more suitable as a biomarker than the cleaved form [10]. Different types of ELISA-based commercial kits are available, but there are variabilities between the kits. More recently, Winnicki et al. compared the performance of two well-known ELISA kits (Quantikine Human uPAR ELISA and suPARnostic™ assay) for kidney disease [64]. They took samples from patients suffering from kidney disease and compared them to healthy controls and found that the cut-off values vary between the two ELISA kits. Similar studies are also warranted in the case of cancer in order to exploit the true diagnostic potential of suPAR.

## 3. Therapeutic Targeting of the Fibrinolytic System

**Figure 1:**
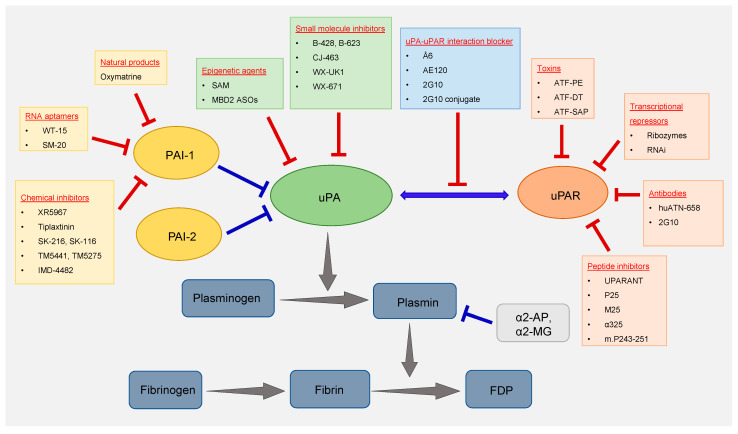
Components of the fibrinolytic system. The drug/inhibitors targeting the key members of the fibrinolytic system (uPA, uPAR, and PAI-1) that are deregulated in cancer are listed inside the colored boxes.

Among the components of the fibrinolytic system, uPA was the first to be targeted for the treatment of cancer. Over the years, different classes of agents were used to target uPA. Some of the most important ones are summarized below and depicted in Figure 1.

### 3.1. Small Molecule Inhibitors of uPA

In 1987, Vassalli et al. reported that amiloride could selectively block the catalytic activity of uPA [65]. Later on, by some modification in the amiloride structure, Towle et al. developed a novel class of small molecule inhibitors that interfered with the catalytic activity of uPA [66]. Further investigations of two compounds (B-428 and B-623) belonging to this class revealed that they possess higher inhibitory effects than amiloride to repress the uPA catalytic activity. Subsequently, work from our group demonstrated that B-428 administration significantly reduced prostate cancer growth and metastasis in vivo without causing any detrimental side effects [67]. We then checked whether single-agent treatment with B-428 could show similar anti-cancer effects in the case of other types of cancer and found that the inhibitor caused a statistically significant reduction in breast tumor volume and metastatic spread into the distant organ in preclinical settings [68]. Furthermore, B-428 in combination with tamoxifen (an approved drug for the treatment of hormone receptor-positive breast cancer) additively reduced mammary tumor volume and distant metastasis in vivo. CJ-463 is another small molecule inhibitor of uPA with an inhibitory constant (*K*_i_) value of 20 nM [69] and significantly reduced tumor volume and metastasis in a murine model of lung cancer [70]. Wilex AG, a biopharmaceutical company, developed several potent uPA inhibitors that went to human clinical trials, where they showed some promising results. WX-UK1, a small molecule uPA inhibitor from Wilex AG, significantly reduced breast cancer growth and metastasis in rodent models [71]. Upamostat (also known as MESUPRON^®^ or WX-671) is a second-generation serine protease inhibitor that targets uPA. Upamostat is a prodrug of WX-UK1 and has shown encouraging results in human clinical trials [72,73,74].

### 3.2. Repression of uPA Gene Expression by Epigenetic Agents

Epigenetic alterations are commonly seen during different malignancies [38], and targeting such abnormalities using epigenetic agents has become an area of immense interest over the last two decades, especially after the approval of the first epigenetic drug Vidaza against hematological cancers [75]. We have previously shown that *uPA* gene expression is increased in cancer because of DNA hypomethylation of its promoter region [41]. DNA methylation is a chemically reversible process [76], and the treatment of cancer cells with the global methyl donor S-adenosylmethionine (SAM) significantly reduces the expression of uPA through the reversal of the hypomethylated state in vitro [77,78,79]. More recently, we showed that oral administration of SAM reduces the tumor volume and metastasis of highly invasive triple-negative MDA-MB-231 breast cancer xenografts implanted into the fat pad of immunocompromised mice [80]. Further analysis of the primary tumors revealed that SAM treatment significantly reduces *uPA* gene expression compared to the vehicle-treated control arm. SAM is a naturally occurring agent with little to no documented toxicity, and such treatment holds promise for future combination studies with different potent anti-cancer agents. Moreover, microarray-based gene expression analyses of MDA-MB-231 breast cancer cells revealed a downregulation of genes involved in the uPA/uPAR pathway upon SAM treatment, suggesting the possible epigenetic modulation of the axis in cancer [80]. SAM is a pleiotropic molecule and utilized as a cofactor by many enzymes, some of which are involved in tumorigenesis. For example, nicotinamide N-methyltransferase (NNMT) uses endogenous SAM to mediate its enzymatic activity [81]. Overexpression of NNMT has been seen in many cancers [82,83,84]. However, there is no direct evidence showing increased NNMT activity upon exogenous SAM treatment to treat cancer. Regardless, more detailed studies are warranted to determine whether exogenous administration of SAM provides survival advantages to the cancer cells. 

We have also shown that targeting methyl-CpG-binding domain protein 2 (MBD2), a protein that can read the DNA methylation marks and is frequently upregulated in many cancers [85], using a 20-mer antisense oligonucleotide (ASO) reduced uPA gene expression in breast and prostate cancer cells [77,78]. 

### 3.3. Transcriptional Repression of uPA and uPAR

Gene therapy-based strategies have been attempted to target the transcription of the uPA or uPAR gene. Karikó et al. synthesized a 37-mer hammerhead ribozyme to target the uPAR mRNA and delivered it into osteosarcoma cells using lipofectaime [86]. They found that the artificially synthesized ribozymes entered into the cytoplasm of cancer cells, cleaved the 1.4 kilobase uPAR mRNA, and thereby caused a dose-dependent decrease in its translation into a protein. 

RNA interference (RNAi) against the uPA/uPAR genes has also been tested. Mohan et al. demonstrated that adenovirus-mediated delivery of an antisense construct targeting the elevated uPAR expression in high-grade glioma markedly reduced tumor growth in vivo [87]. Margheri et al. used an 18-mer ASO against uPAR known as “uPAR aODNs” that significantly reduced uPAR levels and subsequently decreased prostate cancer bone metastases when PC3 cells were injected into immunocompromised animals via intracardiac injections [88]. In SNB19 glioma cells, RNAi-mediated repression of uPA and uPAR gene expression retarded the oncogenic PI3K/AKT/mTOR axis and increased Fas ligand-mediated apoptosis [89]. Even though various attempts have been made to repress uPA and uPAR transcriptionally, none of them made it to clinical trials. One possible reason is that there has been a general reluctance to use “nucleic acid-based therapies” over the years. However, with the global use of mRNA-based vaccines against SARS-CoV-2, “nucleic acid-based therapies” have finally become more acceptable. Therefore, attempts to transcriptionally repress uPA, uPAR, and many other known oncogenes may see a boost in clinical settings in the near future.

### 3.4. Blocking the uPA–uPAR Interaction

Several approaches to block the interaction between uPA and its receptor uPAR have been tested over the years. In 1993, Crowley et al. used an enzymatically inactive uPA analog by mutating the 356th residue of uPA from serine to alanine and found a significant decrease in prostate cancer metastasis [90]. We have previously shown that Å6, a non-competitive inhibitor of the uPA–uPAR interaction, can cause a significant reduction in breast tumor growth and distant metastasis in vivo [91]. Other groups have reported similar anti-cancer therapeutic benefits of Å6 in other types of cancer as a single-agent monotherapy [92,93]. Moreover, when used in combination with other drugs, Å6 could significantly enhance the anti-cancer effects of tamoxifen [94] and cisplatin [95].

Using a phage display library, Duriseti et al. identified the 2G10 antibody that stably binds to the uPAR protein, blocks its interaction with uPA, and significantly suppresses the invasiveness of cancer cells in vitro [96]. The 2G10 antibody has shown promising therapeutic and diagnostic benefits in animal models of breast cancer [50,97]. More recently, Harel et al. conjugated the 2G10 antibody to an antimitotic cytotoxic payload called monomethylauristatin E (MMAE) via a cathepsin B-cleavable linker, RED-244 [98]. The 2G10 conjugate (2G10-RED-244-MMAE) showed an enhanced anti-cancer therapeutic potential to reduce breast tumors compared to the vehicle-treated control and 2G10 monotherapy-treated groups in vivo. Further studies using the 2G10 antibody alone or the conjugate in different types of cancer are warranted. AE120 is a peptide-based inhibitor of uPAR that has the ability to block uPA binding to uPAR and reduce the invasiveness of HEp-3 human epidermoid carcinoma cells [99].

### 3.5. Peptide Inhibitors against uPAR

uPAR is susceptible to cleavage by proteases such as plasmin, uPA, and metalloproteases. The most susceptible cleavage site of uPAR is located in the linker regions between the D1 and D2 domains. The uPAR protein lacking the D1 domain cannot bind to its most canonical binding partners uPA and vitronectin; however, it can bind to the FPRs and functions in cell migration [100]. Several small molecule peptide inhibitors have been developed that block the interaction between uPAR and FPRs to inhibit their internalization. UPARANT (also known as cenupatide) is the most well-known peptide inhibitor of uPAR that competes with N-formyl-Met-Leu-Phe (fMLF) for binding to the FPRs and thereby blocks the VEGF-directed angiogenesis [100,101]. In addition, several other peptide inhibitors against uPAR have been reported which include P25 [102], M25 [103], α325 [104], and m.P243-251 [105].

### 3.6. Antibodies against uPAR

Antibody-based targeted therapies have shown great promise over the last two decades. We have previously shown that a polyclonal rat anti-uPAR antibody causes a significant reduction in primary breast tumor growth and metastasis in preclinical settings [44]. Thereafter, a monoclonal antibody called ATN-658 was developed to target the human uPAR protein, and administration of the ATN-658 antibody significantly reduced the growth, invasiveness, and metastatic ability of prostate cancer cells both in vitro and in vivo [106]. The ATN-658 antibody is now fully humanized, and our recent studies demonstrated that treatment with the humanized ATN-658 (huATN-658) caused a significant reduction in primary breast tumor growth in vivo [107]. Furthermore, when human MDA-MB-231 and bone metastatic variant MDA-BoM-1833 breast cancer cells were implanted into the tibia of immunocompromised animals, the huATN-658 antibody significantly decreased breast tumor-induced skeletal lesions as well as the growth of the tumor cells within the bone microenvironment. Importantly, the anti-cancer effects showed a further enhancement in the group of animals treated with huATN-658 in combination with the Food and Drug Administration (FDA)-approved bisphosphonate zoledronic acid, suggesting the clinical utility of the antibody for human breast cancer patients. The anti-cancer therapeutic potential of ATN-658 has been evaluated in different types of cancer, where the antibody showed equally promising results [108,109]. As mentioned before, 2G10 is another uPAR antibody that has shown anti-cancer therapeutic potential in vivo [97].

### 3.7. Toxin Conjugation to Deliver the Drugs Targeting uPA/uPAR System

The use of toxins to treat cancer dates back to the early nineteenth century when Vautier described the spontaneous regression of tumors in patients with concurrent *Clostridium* infection [110]. Later on, William B. Coley, a physician based in New York, noticed a curative effect of erysipelas (a bacterial infection of the skin) on patients with sarcoma [111]. He later developed a vaccine from the cocktail of two killed bacteria (*Serratia marcescens* and *Streptococcus pyogenes*) known as “Coley’s toxins” for the treatment of various types of cancer [112,113,114]. The earlier success of Dr. Coley’s treatment strategy led to the development of recombinant toxins for use in cancer treatment [115]. The major advantage of toxin-based cancer therapeutics is that the bacteria can be manipulated for a more selective delivery system [110]. When used in combination with conventional standard-of-care cancer therapies, the bacterial toxins may enhance therapeutic response [115,116]. Several toxins targeting the uPA/uPAR system have been assessed for the treatment of different malignancies in the past two decades. The most prominent strategy in this regard has been the conjugation of ATF with a suitable toxin for targeting uPAR-expressing cells. For example, ATF conjugated with a truncated *Pseudomonas* exotoxin (PE) showed significant cytotoxic effects in a panel of well-established cancer cell lines belonging to various malignancies [117]. Vallera et al. demonstrated that conjugation of the catalytic portion of diphtheria toxin (DT) with ATF caused a significant reduction in the proliferation of glioblastoma cells in vitro and reduced tumor volumes in vivo [118]. In another study, a bispecific immunotoxin, DTATEGF, targeting the EGF/EGFR and uPA/uPAR axes showed a potent cytotoxic effect in human metastatic non-small cell lung cancer (NSCLC) brain tumor xenografts [119]. More recently, Zuppone et al. showed that conjugation of a ribosome-inactivating protein called saporin (SAP) with ATF significantly reduced the viability of breast and bladder cancer cell lines [120]. Furthermore, the anti-cancer effects of the ATF–SAP conjugate were selective towards cancer cells with no discernable effect on the growth of non-tumorigenic fibroblast cells expressing high levels of uPAR. Even though the bacterial toxins showed great promise for targeting the uPA/uPAR system, more research is needed before their successful translation to human clinical trials. Bacterial toxins may elicit unwanted immunogenicity and septic shock to the host, which is a major concern regarding their use in humans [115]. 

### 3.8. Chemical Inhibitors of PAI-1

Even though PAI-1 levels are markedly elevated in many types of cancer, therapeutic agents targeting PAI-1 have not been developed to the same extent as uPA/uPAR. The first class of PAI-1 inhibitors was reported in the 1990s [121]; however, they were mostly used for clot lysis rather than cancer therapeutics. XR5967, a diketopiperazine that can block the activity of human and murine PAI-1, significantly reduced cancer cell invasion, migration, and angiogenesis in vitro [122]. Tiplaxtinin (also known as PAI-039) inhibits cell proliferation, colony formation, and angiogenesis and increases apoptosis by blocking PAI-1 expression in several types of cancer [123,124,125]. Oral administration of a specific PAI-1 inhibitor, SK-216, inhibited tumor progression in a murine model of Lewis lung carcinoma [126]. However, in the same study, SK-216 administration did not show any significant effect in reducing B16 melanoma tumor volume in vivo, suggesting a possible cancer-type specificity of PAI-1 inhibition. Mutoh et al. showed that two PAI-1 inhibitors, SK-216 and SK-116, could individually reduce the number of intestinal polyps and thereby function as chemopreventive agents for colorectal cancer [127]. Two orally available small molecule anti-PAI-1 agents, TM5441 and TM5275, inhibited the proliferation of different types of cancer cell lines (MDA-MB-231 (breast cancer), HCT116 (colorectal cancer), HT1080 (fibrosarcoma), Jurkat (acute T cell leukemia), Daoy (medulloblastoma)) with an IC50 range between 9.7 and 60.3 μM [128]. However, the anti-cancer effects were not common for all the cancer types as some cell lines did not respond to TM5441 and TM5275 treatments in vitro [128]. In another study, TM5275 inhibited ovarian cancer cell proliferation in vitro [129]. Another PAI-1 inhibitor, IMD-4482, caused decreased ovarian cancer cell proliferation and invasion and induced apoptosis in vitro [130]. Moreover, IMD-4482 administration could reduce tumor volume and distant metastasis by inhibiting the phosphorylation of focal adhesion kinase (FAK) in vivo.

### 3.9. RNA Aptamers as PAI-1 Inhibitors

RNA aptamers are single-stranded nucleic acids that can tightly bind to specific targets and are used for various diagnostic and therapeutic applications [131]. By using combinatorial chemistry techniques, Blake et al. identified the RNA aptamers SM-20 and WT-15 that bind to PAI-1 with high affinity and specificity and thereby disrupt the interaction of PAI-1 with vitronectin and heparin [132]. The disruption in the PAI-1–vitronectin interaction shows anti-metastatic potential. WT-15 and SM-20 could also reduce the invasiveness and migratory properties of the highly invasive MDA-MB-231 breast cancer cell line in vitro [133]. Further studies are warranted to know whether similar anti-metastatic properties of the RNA aptamers can be replicated in the case of other types of cancer. In addition, preclinical studies using RNA aptamers are also needed to understand whether they show similar anti-cancer effects in vivo.

### 3.10. Natural Products as PAI-1 Inhibitors

Wang et al. showed that treatment of colorectal cancer cells with oxymatrine, a quinolizidine alkaloid derived from the Chinese herb *Sophora flavescens*, caused a significant reduction in cell proliferation and migration [134]. At the molecular level, oxymatrine treatment reduced the expression of PAI-1 and components of the TGF-β signaling. Moreover, oxymatrine induced the expression of the epithelial cell marker (E-cadherin) while decreasing the expression of the mesenchymal marker (α-Smooth muscle actin), which reversed the epithelial-to-mesenchymal (EMT) state and thereby reduced migration. However, the exact mechanism by which oxymatrine downregulates PAI-1 expression is not known and warrants further exploration.

## 4. Conclusions and Future Perspectives

Even though several key components (uPA, uPAR, and PAI-1) of the fibrinolytic system are clearly deregulated in almost all cancers and are potential diagnostic and therapeutic targets, their clinical translation into human cancer patients has been relatively less explored. Some of the earlier attempts to inhibit components of the fibrinolytic system showed a modest response in preclinical settings as the drugs/inhibitors used were not entirely specific against the component. For example, amiloride can target several factors other than uPA, notably, the epithelial sodium channel (ENaC), acid-sensitive ionic channel (ASIC), and ornithine decarboxylase [135,136]. It is possible that the amiloride-mediated anti-cancer effects were seen because of the combined inhibition of several targets. Moreover, it is possible that the tumor cells activate alternative pathways to interfere with the efficacy of the drugs targeting the fibrinolytic system. Such activation of compensatory pathways has been observed for several approved anti-cancer drugs [137,138]. Therefore, detailed studies on the interplay between the various inhibitors of the fibrinolytic system during tumor progression are warranted in the future. With the advent of high-throughput screening technologies, it is now straightforward to assess the genome-wide effects of a particular drug. It will be interesting to utilize such screening methods to evaluate the collateral effects of targeting the various components of the fibrinolytic system. This will also help to design better combination strategies for treating different types of cancer. 

One of the encouraging frontiers that has recently drawn much attention is the utility of uPAR-based oncological imaging. More than ten phase 2 trials assessing the effectiveness of uPAR-based imaging are either ongoing or have been completed so far. suPAR is emerging as a new candidate biomarker for several pathological conditions; however, the studies related to the use of suPAR as a potential biomarker are still at an infancy. More research is warranted in this regard. It remains to be seen whether the strong preclinical evidence of some of the antibodies and small molecule inhibitors against the components of the fibrinolytic system could still be translated in human patients through appropriate double-blinded randomized clinical trials.

## Data Availability

Not applicable.
